# Decreased inhibition of exosomal miRNAs on SARS-CoV-2 replication underlies poor outcomes in elderly people and diabetic patients

**DOI:** 10.1038/s41392-021-00716-y

**Published:** 2021-08-11

**Authors:** Yanbo Wang, Xiaoju Zhu, Xia-Ming Jiang, Jingwei Guo, Zheng Fu, Zhen Zhou, Ping Yang, Hongyuan Guo, Xu Guo, Gaoli Liang, Ping Zeng, Gengfu Xiao, Jizheng Ma, Xin Yin, Lei-Ke Zhang, Chao Yan, Chen-Yu Zhang

**Affiliations:** 1 Nanjing Drum Tower Hospital Center of Molecular Diagnostic and Therapy, Chinese Academy of Medical Sciences Research Unit of Extracellular RNA, State Key Laboratory of Pharmaceutical Biotechnology, Jiangsu Engineering Research Center for MicroRNA Biology and Biotechnology, NJU Advanced Institute of Life Sciences (NAILS), NJU Institute of AI Biomedicine and Biotechnology, Chemistry and Biomedicine Innovation Center (ChemBIC), School of Life Sciences, Nanjing University, Nanjing, China; 2 State Key Laboratory of Virology, Wuhan Institute of Virology, Center for Biosafety Mega-Science, Chinese Academy of Sciences, Wuhan, Hubei China; 3 Department of Clinical Laboratory, The Affiliated Drum Tower Hospital of Nanjing University Medical School, Nanjing, China; 4 The MOH Key Laboratory of Geriatrics, Beijing Hospital, National Center of Gerontology, Institute of Geriatric Medicine, Chinese Academy of Medical Sciences, Beijing, China; 5 The Research Center of Military Exercise Science, The Army Engineering University of PLA, Nanjing, Jiangsu China

**Keywords:** Non-coding RNAs, Infectious diseases

## Abstract

Elderly people and patients with comorbidities are at higher risk of COVID-19 infection, resulting in severe complications and high mortality. However, the underlying mechanisms are unclear. In this study, we investigate whether miRNAs in serum exosomes can exert antiviral functions and affect the response to COVID-19 in the elderly and people with diabetes. First, we identified four miRNAs (miR-7-5p, miR-24-3p, miR-145-5p and miR-223-3p) through high-throughput sequencing and quantitative real-time PCR analysis, that are remarkably decreased in the elderly and diabetic groups. We further demonstrated that these miRNAs, either in the exosome or in the free form, can directly inhibit S protein expression and SARS-CoV-2 replication. Serum exosomes from young people can inhibit SARS-CoV-2 replication and S protein expression, while the inhibitory effect is markedly decreased in the elderly and diabetic patients. Moreover, three out of the four circulating miRNAs are significantly increased in the serum of healthy volunteers after 8-weeks’ continuous physical exercise. Serum exosomes isolated from these volunteers also showed stronger inhibitory effects on S protein expression and SARS-CoV-2 replication. Our study demonstrates for the first time that circulating exosomal miRNAs can directly inhibit SARS-CoV-2 replication and may provide a possible explanation for the difference in response to COVID-19 between young people and the elderly or people with comorbidities.

## Introduction

COVID-19, caused by the SARS-CoV-2 virus, is a virulent pneumonia with >180,000,000 confirmed cases worldwide and >4,000,000 deaths as of July 2021.^1^ An epidemiologic study suggested that elder people and patients with comorbidities, such as diabetes, are more susceptible to COVID-19 infection and the development of more severe disease,^2–4^ a feature shared with the 2003 SARS epidemic.^5^ Chinese researchers first reported a higher prevalence of diabetes among patients with severe compared to non-severe illness.^6^ In the initial studies, diabetes patients showed to be 2.26 times more common in patients with more severe COVID-19 compared to those with less severe infection, while at the same time the presence of diabetes entailed an odds ratio of 2.85 of intra-hospital mortality.^7^ COVID-19 patients with diabetes are admitted more often to intensive care units (ICUs) compared to those without diabetes,^8^ and the risk of developing severe COVID-19 is higher in people with diabetes.^9^ The worse the glycemic control, the worse the severity of infection and the greater the risk of mortality.^9^ Therefore, understanding the potential mechanism underlying the increased susceptibility of elder people and patients with comorbidities to COVID-19 infection is critical for investigating the pathogenesis and estimating the expected global disease burden.

The immune system protects the body from constant attacks by viruses, bacteria and other pathogens. Much of the protection is provided by immune cells. However, immune functions are not restricted to these “specialists”.^10^ Several lines of evidence indicate that RNA interference (RNAi) plays a role in the antiviral immunity of invertebrates, such as *C. elegans* and *D. melanogaster*.^11,12^ MicroRNAs (miRNAs), a class of non-coding RNAs that are around 22 nucleotides in length, are part of the RNAi system and might also function as an antiviral mechanism in mammals.^13^ miRNAs have been found to interact with viral genes in several ways. For example, miR-32 restricts the accumulation of the retrovirus primate foamy virus type 1 (PFV-1) in human cells.^14^ It has also been reported that mice deficient in Dicer-1 (and therefore deficient in mature miRNAs) are more susceptible to vesicular stomatitis virus (VSV) infection.^15^ Liu et al. calculated the potential miRNA targets in 17 metazoan and viral genomes and proposed that the initial function of miRNAs was predominantly antiviral,^16^ as evolution proceeded, miRNAs acted more specifically on self-genomes, suggesting that the origin of microRNAs is a defensive rather than a regulatory strategy.

Previous studies by our group and others have shown that extracellular miRNAs are highly stable and can not only serve as biomarkers for various diseases but also be secreted into the extracellular space within exosomes.^17–19^ Evidences also exist that these exosomes can be taken up by neighbouring or distant cell and subsequently modulate the function of recipient cells.^18,20,21^ Herein, we tested the hypothesis that circulating miRNAs in exosomes may act as a natural antiviral mechanism to suppress SARS-CoV-2 replication. We provide evidence that several circulating exosomal miRNAs, which were decreased in elder and diabetic people, could inhibit SARS-CoV-2 replication by directly targeting the S protein. Long-term exercise could increase the expression of these exosomal miRNAs and enhance the inhibitory effect on SARS-CoV-2 replication.

## Results

### Circulating miR-7, 24, 145 and 223, which are comparatively lower in elderly people and people with diabetes, directly inhibited SARS-CoV-2 replication

**Fig. 1:**
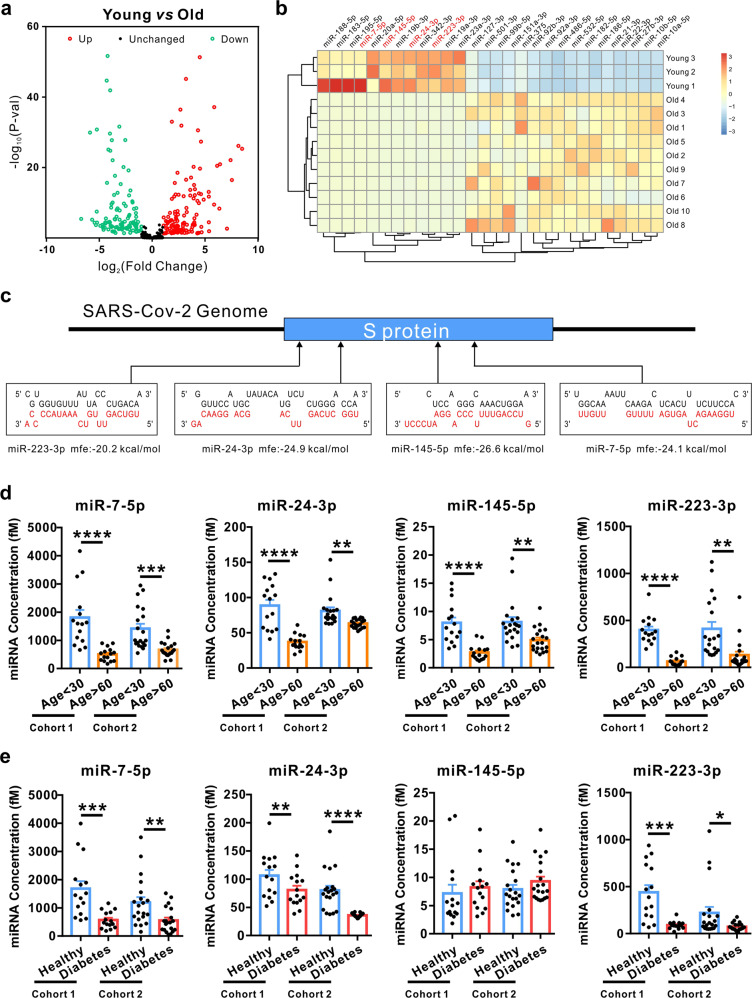
Circulating miR-7, 24, 145 and 223 were decreased in elderly people and people with diabetes. **a** Scatter plot comparison illustrating circulating miRNAs that are differentially expressed between the young and old groups. For comparison of miRNAs, reads were normalized to reads per million (RPM). The analysis of differentially expressed miRNAs used a stringent threshold and a significance criterion of *P* < 0.05 and log2-fold change > 1 (young group: *n* = 3; old group: *n* = 10). **b** Hierarchical clustering indicates significant differences in miRNA expression profiling between the young and elderly groups with stricter criteria of mean reads >50, *P* < 0.01 and fold change > 10 (young group: *n* = 3; old group: *n* = 10). **c** Schematic diagrams of the predicted interaction between upregulated miRNA in the young group and S protein in the SARS-CoV-2 genome. The miRNA sequences are indicated in red. **d**, **e** The expression of four serum miRNAs in individual serum samples. Serum samples from 15 young people and 15 elderly people (**d**), 15 healthy people and 15 diabetic patients (**e**) were collected from The Affiliated Drum Tower Hospital of Nanjing University (Cohort 1) and 20 young people and 20 old people (**d**), 20 healthy people and 20 diabetic patients (**e**) were collected from Nanjing Jiangning Hospital (Cohort 2). All samples were subjected to qRT-PCR for absolute quantification. Data are shown as means ± SEMs. Statistical significance was assessed using the two-tailed unpaired Student’s *t*-test. *P < 0.05; **P < 0.01; ***P < 0.001; ****P < 0.0001

To comprehensively investigate the differences in circulating miRNA expression patterns in the serum between young and elderly people, we first downloaded age-related non-coding RNA expression profiles obtained by high-throughput sequencing from the NCBI Gene Expression Omnibus (GEO) database. A total of 13 samples (3 young, age <30 and 10 old, age >60) were analysed (GSE53439 and GSE111754). Differentially expressed miRNAs were screened and identified by DESeq2. Among the 1202 miRNAs analysed, 114 miRNAs were significantly lower and 126 were significantly higher (fold change > 2 and *P* value < 0.05) in the elderly group compared to the young group (Fig. 1a). We then applied more stringent filters to the screen, only miRNAs that satisfied the following three criteria were considered as significantly differentially expressed: counts per million of total aligned reads (CPM) > 50 in serum by sequencing detection, fold change >10 and *P* value < 0.01 between the two groups. This analysis resulted in 28 significantly differentially expressed miRNAs in the elderly group compared with the young group (Fig. 1b).

To evaluate whether circulating miRNAs can function as antiviral molecules, we used a target prediction tool for miRNAs, RNAhybrid, to analyse potential target sites in the SARS-CoV-2 genome. Bioinformatics prediction showed that 125 of the 240 differentially expressed miRNAs between the young and elderly groups could directly target the genome of SARS-CoV-2 (Table S4). Among them, miR-223-3p, miR-24-3p, miR-145-5p and miR-7-5p were predicted to directly bind to the spike glycoprotein (S protein, the protein that binds to its receptor and mediates membrane fusion and entry of SARS-CoV-2) (Fig. 1c). Next, we used RT-qPCR to determine the levels of the four miRNAs (miR-7-5p, miR-24-3p, miR-145-5p and miR-223-3p) in individual serum samples from the young group (*n* = 15, age < 30) vs elderly group (*n* = 15, age > 60) and from a healthy group (*n* = 15) vs diabetic group (*n* = 15) (Cohort 1). Absolute quantification showed that the levels of all four miRNAs were significantly lower in serum from the elderly group compared with the young group (Fig. 1d). Moreover, three out of the four miRNAs (miR-7-5p, miR-24-3p and miR-223-3p) were also lower in the diabetic group compared with the healthy group (Fig. 1e). We also collected the second cohort of serum samples including the young group (*n* = 20, age < 30) vs elderly group (*n* = 20, age >60) and healthy group (n = 20) vs. diabetic group (*n* = 20) from Nanjing Jiangning Hospital hospital (Cohort 2), and checked the levels of the four miRNAs in individual serum samples. Similar to the data generated from cohort 1, the results showed that all four miRNAs were significantly lower in the serum from the elderly group compared with the young group (Fig. 1d), and three out of the four miRNAs (miR-7-5p, miR-24-3p and miR-223-3p) were also lower in the diabetic group compared with the healthy group (Fig. 1e). These findings indicated that decreased circulating miRNAs in elderly people or people with diabetes may influence SARS-CoV-2 replication by targeting the S protein.

**Fig. 2:**
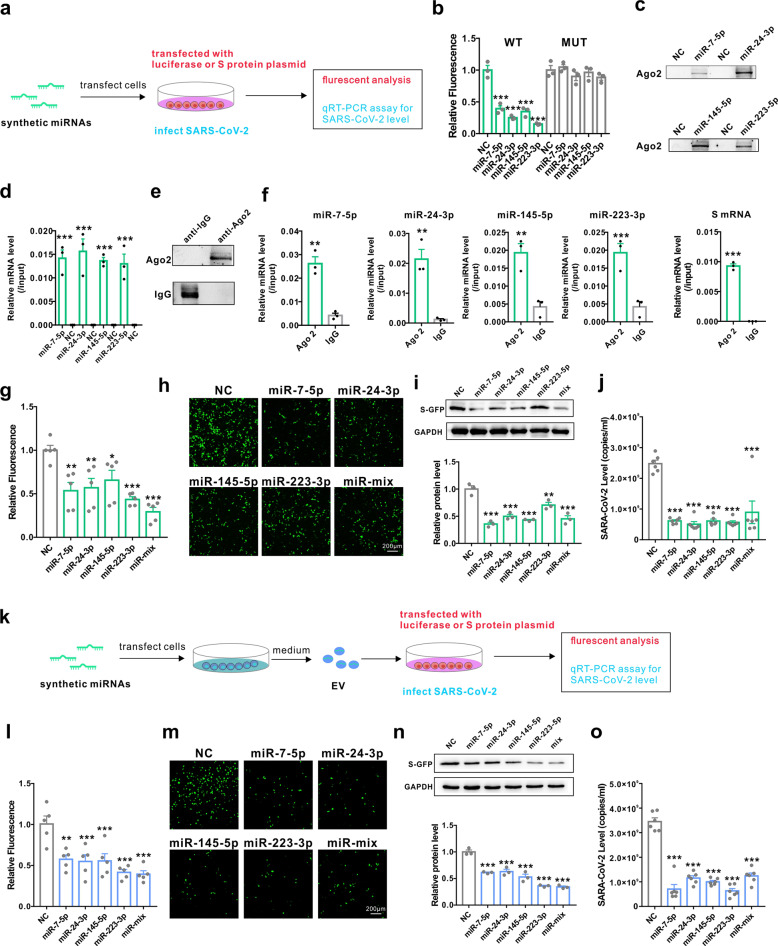
miR-7, 24, 145 and 223 in exosomes directly targeted the S protein and inhibited SARS-CoV-2 replication. **a** Diagrams of the transfection of synthetic miRNA mimics and measurement of the inhibition of S protein expression and antiviral activity in SARS-CoV-2 replication. **b** Luciferase reporter assays in HEK293T cells transfected with reporter vectors carrying the WT binding site sequence of miR-7-5p, miR-24-3p, miR-145-5p or miR-223-3p vs the mutated binding site sequence in the presence or absence of the indicated miRNA mimics. **c** Quantitative analysis of Ago2 protein levels by western blot in HEK293T cells after pulling down with biotinylated control probe (NC), miR-7-5p, miR-24-3p, miR-145-5p or miR-223-3p probe. **d** Quantitative RT-PCR analysis of S mRNA levels in HEK293T after pulling down with biotinylated control probe (NC), miR-7-5p, miR-24-3p, miR-145-5p or miR-223-3p probe. **e** Quantitative analysis of Ago2 or IgG protein levels in anti-Ago2 or anti-IgG antibody-immunoprecipitated complex by western blot. **f** Detection of miR-7-5p, miR-24-3p, miR-145-5p or miR-223-3p and S mRNA in anti-Ago2 or anti-IgG antibody-immunoprecipitated complex by RT-qPCR. **g**, **h**, **i** Cytometric analysis (**g**), fluorescence images (**h**) and western blot (**i**) of GFP-S protein-overexpressing HEK293T cells after treatment with synthetic miR-7-5p, miR-24-3p, miR-145-5p or miR-223-3p individually or in combination (miR-mix). **j** Efficacy of synthetic miR-7-5p, miR-24-3p, miR-145-5p and miR-223-3p in inhibiting the replication of SARS-CoV-2. **k** Diagrams of the collection of exosomes from cell medium after transfection of synthetic miRNA mimics and measurement of the inhibition of S protein expression and antiviral activity in SARS-CoV-2 replication. **l**, **m**, **n** Cytometric analysis (**l**), fluorescence images (**m**) and western blot (**n**) of GFP-S protein-overexpressing HEK293T cells after treatment with exosomes from cells transfected with synthetic miR-7-5p, miR-24-3p, miR-145-5p or miR-223-3p individually or in combination. **o** Efficacy of exosomal miR-7-5p, miR-24-3p, miR-145-5p and miR-223-3p in inhibiting the replication of SARS-CoV-2. Data are shown as means ± SEMs. Statistical significance was assessed using one-way ANOVA coupled with Bonferroni’s post hoc test (**b**, **g**, **i**, **j**, **l**, **n**, **o**), or two-tailed unpaired Student’s *t*-test (**d**, **f**). *P < 0.05; **P < 0.01; ***P < 0.001

We then explored whether circulating miRNAs can act as antiviral agents to suppress SARS-CoV-2 replication. We first generated reporter constructs containing the mRNA of S protein and co-transfected them with synthetic control non-coding RNA (NC), miR-7-5p, miR-24-3p, miR-145-5p and miR-223-3p mimics (Fig. 2a). Co-transfection with mimics of all four miRNAs effectively reduced luciferase activity in HEK293T cells compared to NC (Fig. 2b). Moreover, mutations in the predicted miRNA binding sites completely rescued the reduction in luciferase induced by miRNA mimics, confirming the specificity of the miRNA target sites (Fig. 2b). To further explore whether the four miRNAs could directly bind to S mRNA or Ago2, we designed miR-7-5p, miR-24-3p, miR-145-5p and miR-223-3p mimics whose 3′ terminal was biotinylated (miRNA probe). After transfecting the miRNA probes into HEK293T cells for 24 h, we used streptavidin-coated magnetic beads to pull down biotinylated miRNA and measured the level of co-precipitated Ago2 protein and S mRNA. Both Ago2 protein and S mRNA were enriched in the pull-down product precipitated by all four miRNA probes but not by the non-specific control probe (Fig. 2c, d). Furthermore, we performed co-immunoprecipitation assays to assess the binding between miRNA, mRNA and Ago2. HEK293T cells were co-transfected with miR-7-5p, miR-24-3p, miR-145-5p and miR-223-3p mimics and the S overexpression plasmid for 24 h, Ago2 or IgG protein in the cell lysate were then immunoprecipitated by anti-Ago2 or anti-IgG antibody. The levels of Ago2 protein, Ago2-bound miRNAs and mRNA were assessed by western blot or RT-qPCR. As shown in Fig. 2e, f, Ago2 protein, four miRNAs and the mRNA of S were enriched in the complex immunoprecipitated by the anti-Ago2 antibody but not normal IgG. These results proved that the miRNA-mRNA interaction occurred intra cell involving the Ago2 complex. To further validate the correlation between the four miRNAs and S protein expression, we assessed the effect of the miRNA mimics, either individually or in combination, on the levels of S protein in HEK293T cells overexpressing the GFP-S protein. The expression of S protein in HEK293T cells was significantly inhibited by the introduction of miR-7-5p, miR-24-3p, miR-145-5p and miR-223-3p mimics (Fig. 2g, h and i). Furthermore, these miRNA mimics also significantly inhibited SARS-CoV-2 replication (Fig. 2j). To directly assess the effects of exosomal miRNAs on SARS-CoV-2 replication, exosomes were isolated from the culture medium of HEK293T cells transfected with miR-7-5p, miR-24-3p, miR-145-5p and miR-223-3p mimics and incubated with cells expressing S protein or with SARS-CoV-2 (Fig. 2k). As shown in Fig. 2l, m and n, exosomes containing either of the four miRNAs remarkably inhibited S protein expression as well as SARS-CoV-2 replication (Fig. 2o). These results suggested that miR-7, 24, 145 and 223 in exosomes could directly inhibit S protein expression and SARS-CoV-2 replication.

### The inhibitory effects of circulating exosomes on SARS-CoV-2 replication were decreased in elderly people and diabetic patients

**Fig. 3:**
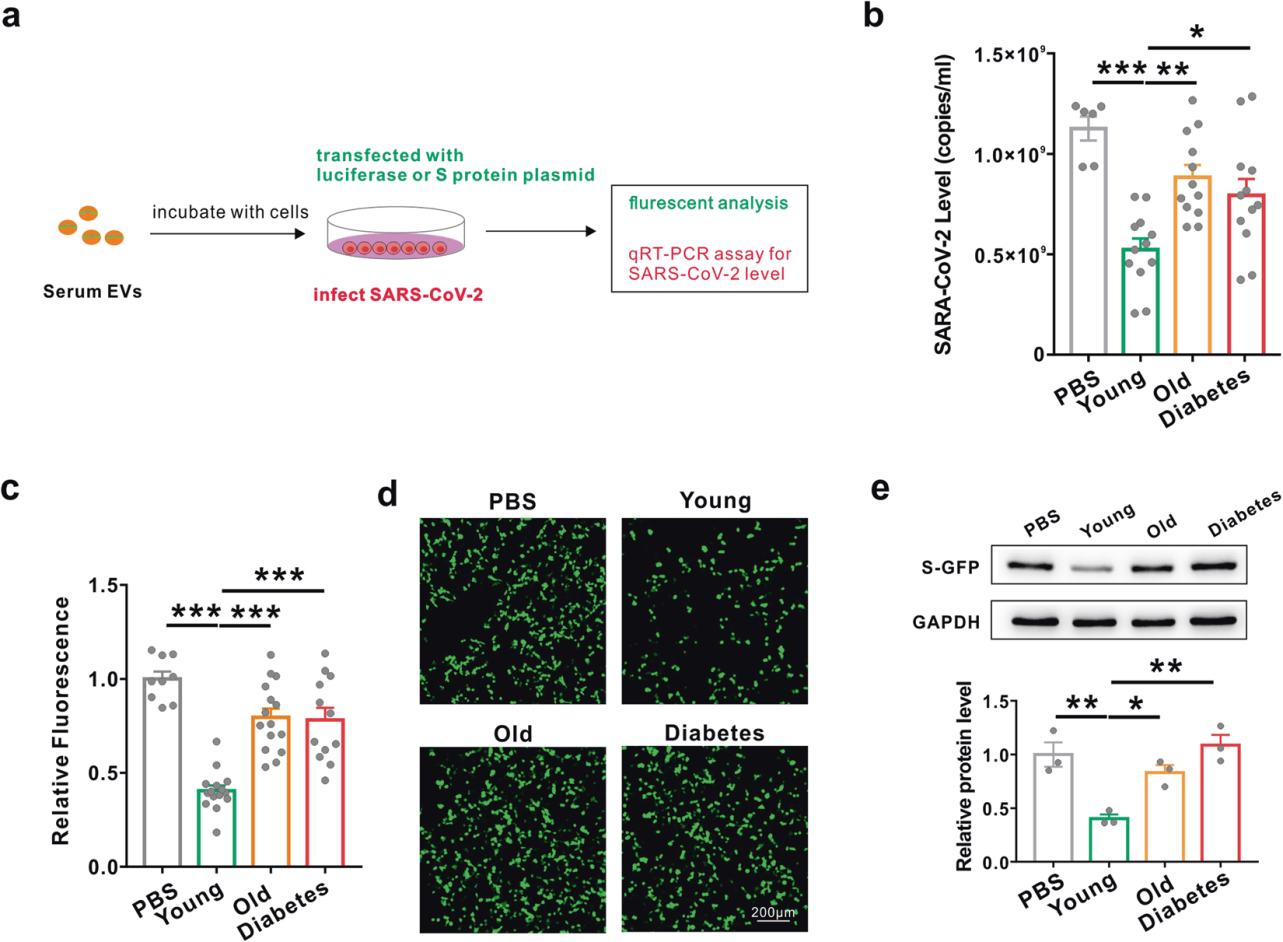
The inhibitory effects of circulating exosomes on SARS-CoV-2 replication were decreased in elderly people and diabetic patients. **a** Diagrams of the collection of exosomes from serum in young, elderly and diabetic groups and the measurement of the biological activities of exosomes in inhibiting S protein expression and SARS-CoV-2 replication. **b** Efficacy of exosomes collected from young, elderly and diabetic groups in inhibiting the replication of SARS-CoV-2. **c**, **d**, **e** Cytometric analysis (**c**), fluorescence images (**d**) and western blot (**e**) of GFP-S protein-overexpressing HEK293T cells after treatment with PBS and serum exosomes collected from the young, elderly and diabetic groups. Data are shown as means ± SEMs. Statistical significance was assessed using one-way ANOVA coupled with Bonferroni’s post hoc test (**b**, **c**, **e**). *P < 0.05; **P < 0.01; ***P < 0.001

Circulating miRNAs in exosomes can be delivered into recipient cells, where they play similar roles as endogenous miRNAs by regulating multiple target genes or signalling events. Based on this, we hypothesized that circulating miRNAs packaged in exosomes could function as extracellular molecules to inhibit SARS-CoV-2 replication. Therefore, circulating exosomes were isolated from sera collected from the young group (age < 30), the elderly group (age > 60) and the diabetic group, and incubated with cells infected with SARS-CoV-2 or S protein (Fig. 3a). As shown in Fig. 3b, circulating exosomes from the young group remarkably inhibited SARS-CoV-2 replication, while exosomes from the elderly and the diabetic groups showed no inhibitory effects. Furthermore, exosomes from the young group showed a similar inhibitory effect on S protein expression, whereas exosomes from the elderly and the diabetic groups showed no effect (Fig. 3c, d and e). These results indicated that circulating exosomes could inhibit SARS-CoV-2 replication and these inhibitory effects are attenuated in both elderly people and diabetic patients.

### Long-term exercise increased the levels of the four antiviral circulating miRNAs and enhanced their inhibitory effect on SARS-CoV-2 replication

**Fig. 4:**
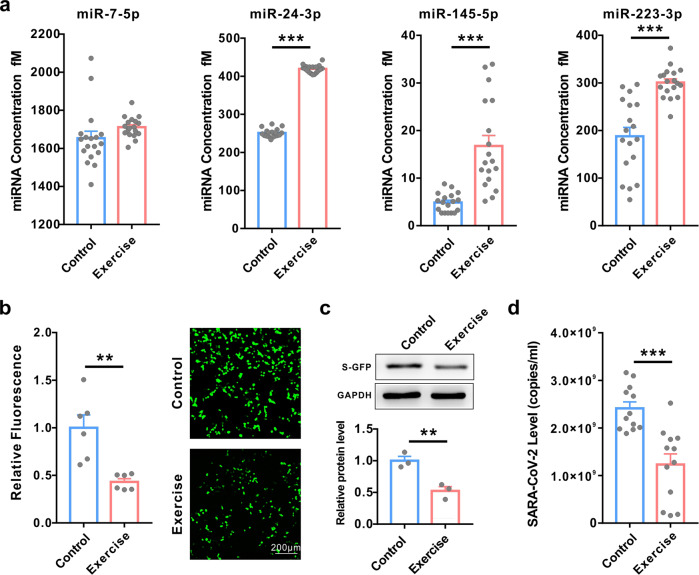
Long-term exercise increased the levels of the four circulating miRNAs and enhanced the inhibitory effects on SARS-CoV-2 replication. **a** Absolute quantification of the expression of the four serum miRNAs in individual serum samples from healthy volunteers with or without 8 weeks of continuous integrative physical exercise (control vs exercise) using qRT-PCR. **b**, **c** Cytometric analysis (**b**, left), fluorescence images (**b**, right) and western blot (**c**) of GFP-S protein-overexpressing HEK293T cells after treatment with serum exosomes collected from healthy people with or without 8 weeks of continuous integrative physical exercise (control vs exercise). **d** Efficacy of exosomes collected from the control and exercise groups in inhibiting the replication of SARS-CoV-2. Data are shown as means ± SEMs. Statistical significance was assessed using the two-tailed unpaired Student’s *t*-test. **P < 0.01; ***P < 0.001

Exercise has many benefits, such as improvements in muscle mass, body composition, physical function, bone mineral density, insulin sensitivity, and cardiovascular health, all of which can help to prevent or delay ageing and diabetes development.^22,23^ Our previous studies have reported that variable of circulating miRNAs is dynamically changing during body exercise. Moreover, the nature and magnitude of the responses of the circulating miRNAs were influenced by exercise type, which likely depends on exercise intensity or volume.^24–26^ Thus, we hypothesized that exercise may also help to inhibit SARS-CoV-2 replication through regulating circulating miRNAs profiles. To investigate whether exercise can improve the ability to resist SARS-CoV-2, we first measured the levels of the four circulating miRNAs in individual serum samples from healthy volunteers with or without 8-weeks’ continuous physical exercise. Absolute quantification showed that three out of the four miRNAs (miR-24-3p, miR-145-5p and miR-223-3p but not miR-7-5p) were significantly increased in serum from the long-term exercise group compared with the control group (Fig. 4a). Next, circulating exosomes from serum samples with or without 8-weeks’ continuous integrative physical exercise were isolated and incubated with cells expressing S protein or with SARS-CoV-2. As shown in Fig. 4b, c, the circulating exosomes from the long-term exercise group remarkably inhibited S protein expression compared with those from the control group. Furthermore, exosomes from the long-term exercise group also significantly inhibited SARS-CoV-2 replication compared with the control group (Fig. 4d). These results indicated that long-term exercise could enhance the inhibitory effects on SARS-CoV-2 replication through upregulation of the antiviral miRNAs in the serum.

## Discussion

Infected patients with old age and comorbidities (diabetes, chronic renal disease and/or chronic pulmonary disease) have higher risks of developing severe complications and higher mortality from COVID-19.^2–4^ However, the underlying mechanisms are unclear. In this study, we first identified 240 differentially expressed circulating miRNAs between the young and elderly people and predicted that 125 of the 240 miRNAs could directly target the genome of SARS-CoV-2 (Table S4). A more stringent screening narrowed down the candidates to four miRNAs (miR-7-5p, miR-24-3p, miR-145-5p and miR-223-3p), all of which are downregulated in elderly people and predicted to target S protein of SARS-CoV-2. Similar results were observed in diabetic patients compared to healthy controls. Furthermore, we demonstrated that both the synthetic free form and exosomal packaged miR-7, 24, 145 and 223 could directly inhibit S protein expression and SARS-CoV-2 replication. Although other and our previous studies have reported that exogenous plant or mesenchymal stem cell-derived miRNAs could directly inhibit SARS-CoV-2,^27–30^ our results demonstrated for the first time that our own endogenous miRNAs could directly inhibit the SARS-CoV-2 virus. This is in accordance with our previous studies showing that approximate 89% (599) of the 675 viruses that infect humans are significantly targeted by human miRNAs.^16^ This new study, together with previous reports of ours and other groups, strongly suggest that miRNAs may serve as an antibody-like endogenous natural antiviral molecule. Understanding the role of miRNAs in antiviral function provides a new perspective for exploring the pathological mechanism of the susceptibility of the elderly and diabetic people to COVID-19 infection.

From the therapeutic point of view, our data showed that exosomes from the serum of young people can significantly inhibit SARS-CoV-2 infection and reduce viral load to ~50%. These findings provided a rationale for using serum/plasma or isolated exosomes from young people to treat patient with COVID-19. Furthermore, as shown in Fig. 4, three circulating antiviral miRNAs were all greatly increased in serum from healthy volunteers after 8 weeks of continuous integrative physical exercise. Circulating exosomes isolated from these individuals also showed stronger inhibitory effects on S protein expression and SARS-CoV-2 replication. These findings suggest that long-term continuous exercise may be an effective way to prevent and fight SARS-CoV-2.

In summary, we defined a distinctive circulating miRNA signature in elderly people by high-throughput RNA sequencing followed by individual qRT-PCR evaluation. We provided evidences that several circulating miRNAs, which are decreased in elderly people and diabetic patients, could inhibit SARS-CoV-2 replication by directly targeting the S protein. We also demonstrated that serum exosomes could inhibit SARS-CoV-2 replication, and this inhibitory effect was weakened in elderly people and diabetic patients. Long-term exercises could increase the expression of these antiviral miRNAs in blood and promote the inhibitory effects on SARS-CoV-2 replication. These results may help us to better understand the COVID-19 disease and provide feasible strategies for both diagnosis and treatment of COVID-19 in the clinic.

## Materials and methods

### Human serum samples

Serum samples from the young group (*n* = 15, age < 30), elderly group (*n* = 15, age > 60), healthy group (*n* = 15) and diabetic group (*n* = 15) were collected from The Affiliated Drum Tower Hospital of Nanjing University (Cohort 1); another cohort of serum samples including the young group (*n* = 20, age < 30), old group (*n* = 20, age > 60), healthy group (*n* = 20) and diabetic group (*n* = 20) were collected from Nanjing Jiangning Hospital (Cohort 2). For the long-term exercise study, serum was collected from a total of 36 healthy male cadets at the College of Basic Education for Commanding Officers, China. All participants underwent a complete physical examination and an electrocardiogram to determine their physical and mental conditions. In the exercise study, all participants were found healthy and were randomly divided into the control group and the exercise group. The exercise group underwent an 8-weeks integrative training programme, which was based on the previous recommendations for optimizing the adaptations to integrative training, including performing high-intensity endurance training sessions first^31,32^ and at least 24 h of recovery between endurance and strength training. Written informed consent was obtained from each individual before enrolment. All the samples were collected by using the same standard operating methods and at the same time period (8:00–9:00 am and the date difference between control and experimental group was no more than 1 week). The general characteristics of all the participants are described in Tables S1 and S2. This study was conducted in accordance with the Declaration of Helsinki and approved by the institutional review boards of all participating institutions.

### Eight-weeks integrative training

The 8-weeks integrative training programme was mainly based on previous reports.^31,32^ All participants were randomly divided into the control group and the exercise group. Participants in exercise group were subjected to Integrative Training for a period of 8 weeks (detailed protocol is listed in Table S3). Briefly, participants were subjected to three times of the following exercise during every week: cardiorespiratory exercise training (participants carried 20 kg of weight for 100–120 min in the field environment); muscle maximal strength and endurance training (the muscle strength protocol includes six sets of 6 repetitions at 90% of 1RM intensity with a 3-min rest interval between exercises and sets, the muscle endurance protocol includes six sets of 20 repetitions at 40% of 1RM intensity with a 1-min rest interval between exercises and sets); plyometric training (this training includes single- or double-leg frontward barrier hop, lateral barrier hop, skip and backward skip, the distance was 20 m, and the hurdle barrier height was 40 cm, each exercise was performed in three sets with a 5-min rest interval between exercises and sets); anaerobic exercise training and stability training (the anaerobic exercise includes 600-m all-out run for three times with a 5-min rest interval between exercises, stabilization exercises includes the bridge, unilateral bridge, side bridge and plank for three sets of 1.5–3 min duration with a 5-min rest interval between exercises).

### Cell lines and virus

The HEK293T cell line and African green monkey kidney Vero E6 cell line were obtained from American Type Culture Collection (ATCC) and maintained in DMEM (Gibco, Carlsbad, CA, USA) with 10% FBS (Gibco) at 37 °C with 5% CO_2_. A SARS-CoV-2 clinical isolate (nCoV-2019BetaCoV/Wuhan/WIV04/20191) was propagated in Vero E6 cells. All experiments with live SARS-CoV-2 were conducted in a biosafety level 3 (BSL3+) facility.

### Exosome isolation

Exosomes were isolated from the serum and cell culture medium as previously described.^33–35^ Briefly, after discarding cell debris by centrifugation at 300 × *g*, 1200 × *g* and 10,000 × *g*, the supernatant was centrifuged at 110,000 × *g* for 70 min (all steps were performed at 4 °C). The pellet containing the exosome was collected and resuspended in PBS.

### Small RNA sequencing assay

To investigate circulating miRNA expression patterns in serum from young or elderly people, we downloaded non-coding RNA profiles obtained by high-throughput sequencing from the NCBI GEO database (GSE53439 and GSE111754). A total of 13 samples (3 young, age < 30 and 10 old, age > 60) were analysed. The differentially expressed miRNAs were screened and identified by DESeq2.

### miRNA target prediction and luciferase assay

The SARS-CoV-2 genome sequence was obtained from the NCBI database (RefSeq ID: MN908947). The target prediction tool RNAhybrid was employed to predict potential miRNA binding sites on the SARS-COV-2 genome. Target sequences were synthesized and subcloned into a luciferase reporter vector (GenScript, Nanjing, China). To test binding specificity, sequences that interacted with the miR-7-5p, miR-24-3p, miR-145-5p and miR-223-3p seed sequence were, respectively, mutated from TCTTCCA to AGAAGGT, CUCCCACCA to GAGGGTGGT, AAACTGGA to TTTGACCT and CUGACA to GACTGT, and the synthetic mutant fragment was inserted into an equivalent reporter plasmid. HEK293T cells were seeded in triplicate in 24-well plates and co-transfected with 50 pmol synthetic scrambled RNA (NC, random sequences and 20–22 nt in length) or miRNA (GenePharma, Shanghai, China) and 0.2 µg per well WT or mutant plasmid. At 24 h post-transfection, cells were lysed, and luciferase activities were measured using the Luciferase Assay Kit (Promega, Madison, WI, USA).

### Overexpression and quantification of spike protein

To overexpress Spike protein, the plasmid (2019-nCov_pcDNA3.1 (+)-P2A-GFP) was purchased from GenScript (Nanjing, China) without codon optimization. After transfection of the plasmid in HEK293T cells, the S-GFP fusion protein was assessed by western blotting using antibody against GFP (Abcam, Cambridge, UK). GAPDH (Santa Cruz Biotechnology, Inc., Santa Cruz, CA, USA) served as an internal control.

### Pull-down assay

miRNA pull-down was performed as described previously.^36^ Briefly, HEK293T cells were co-transfected with both S protein plasmid and single biotinylated miR-7-5p, miR-24-3p, miR-145-5p, miR-223-3p (miRNA probe) or control probe (NC, Genescript, Nanjing, China). The cells were harvested in lysis buffer (20 mM Tris pH 7.5, 100 mM KCl, 5 mM MgCl_2_, 0.5% NP-40 and 1 U/ul Recombinant RNAse inhibitor (TaKaRa)), and total RNA was pretreated with DNaseI and then heated at 65 °C for 5 min, followed by an instant ice bath. Then the RNA was incubated with streptavidin-coated magnetic beads (New England BioLabs, S1420S) at 4 °C for 4 h, with constant rotation. After incubation, two washes with lysis buffer were performed, and protein or RNA was extracted with RIPA or TRIzol and subjected to western blot or qRT-PCR analysis separately.

### Co-immunoprecipitation

Co-immunoprecipitation was performed as described previously.^33^ Briefly, HEK293T cells were co-transfected with both S protein plasmid and a mixture of miR-7-5p, miR-24-3p, miR-145-5p, miR-223-3p mimics. After 24 h, cells were then extensively washed with ice-cold PBS, collected and lysed with 150 μl of lysis buffer (20 mM Tris-HCl, 150 mM NaCl, 0.5% Nonidet P-40, 2 mM EDTA, 0.5 mM DTT, 1 mM NaF, 1 mM PMSF and 1% Protease Inhibitor Cocktail (Sigma), pH 7.5). Lysates were harvested at 16,000 × *g* (10 min, 4 °C) and immunoprecipitated with a rabbit anti-Ago2 antibody or normal IgG followed by protein G Sepharose beads. After elution from the beads, protein or RNA was extracted with RIPA or TRIzol and subjected to western blot or qRT-PCR analysis separately.

### Evaluation of the inhibitory effect of synthetic miRNAs and exosomes on the S protein

To test the inhibitory effect of exosomes on S protein, HEK293T cells were transfected with GFP-S protein plasmid by using Lipofectamine 2000 and then incubated with exosomes isolated from 6 ml of cell medium or 200 μl of human serum. At 18 h post-treatment, fluorescent and cytometric analyses were performed to test the expression level of the GFP-S protein.

To test the inhibitory effect of synthetic miRNAs on the S protein, HEK293T cells were co-transfected with GFP-S protein plasmid (GenScript) and synthetic miRNA or scrambled RNA by using Lipofectamine 2000. At 18 h post-treatment, fluorescent and cytometric analyses were performed to test the expression level of GFP-S protein.

### Evaluation of antiviral activities of synthetic miRNAs and exosomes

To test the antiviral efficacy of exosomes from human serum or cell culture medium, Vero E6 cells were cultured overnight in a 48-well Petri dish with a density of 5 × 10^4^ cells/well. Cells were pretreated with exosomes isolated from 2 ml of cell medium or 62.5 μl of serum for 8 h. To test the antiviral efficacy of synthetic miRNAs, Vero E6 cells were cultured overnight in a 48-well Petri dish with a density of 5 × 10^4^ cells/well. Cells were transfected with synthetic miRNA mimics or scrambled RNA for 8 h. Subsequently, treated Vero E6 cells were infected with the SARS-CoV-2 clinical isolate (nCoV-2019BetaCoV/Wuhan/WIV04/2019) at a multiplicity of infection (MOI) of 0.01. After 1 h of incubation, the virus-exosome mixture was removed, and the cells were washed with warm PBS and incubated in a fresh medium. At 24 h post-infection, the cell supernatant was collected and lysed. Viral RNA extraction and quantitative real-time PCR (RT-PCR) analysis were described in our previous study.^37^

### RNA isolation and quantitative RT-PCR assays

Total RNA was extracted from 100 μl serum or exosomes and dissolved in 20 μl DEPC water, as described previously.^33^ To detect miRNA levels, quantitative RT-PCR was performed using TaqMan miRNA probes (Applied Biosystems, CA, USA) using an LC96 PCR machine according to the manufacturer’s instructions. For the absolute quantitative analysis of miRNAs, a series of synthetic miRNA oligonucleotides of known concentrations were reverse transcribed and amplified to generate a standard curve. The absolute amount of miRNA was then calculated about the standard curve.

### Data statistics

All statistical tests were performed using GraphPad Prism software 7 (San Diego, CA, USA). Data are presented as means ± SEMs. Differences were considered statistically significant at *P* < 0.05. Normality and equal variances among groups were assessed using the Shapiro–Wilk test and Brown–Forsythe tests, respectively. When normality and equal variance were achieved between sample groups, one-way ANOVA (followed by Bonferroni’s multiple comparisons test) or *t*-test was used.

## Supplementary information

Supplementary Materials

Supplementary table 4

## Data Availability

Quantitative data that support the findings of this study are available within the paper and the supplementary Data Set. All other data that support the findings of this study are available from the corresponding author on reasonable request.

## References

[1] WHO. *WHO Main Website*, https://www.who.int (2020).

[2] Davies NG (2020). Age-dependent effects in the transmission and control of COVID-19 epidemics. Nat. Med..

[3] Zhou F (2020). Clinical course and risk factors for mortality of adult inpatients with COVID-19 in Wuhan, China: a retrospective cohort study. Lancet.

[4] Docherty AB (2020). Features of 20133 UK patients in hospital with covid-19 using the ISARIC WHO Clinical Characterisation Protocol: prospective observational cohort study. BMJ.

[5] Anderson RM (2004). Epidemiology, transmission dynamics and control of SARS: the 2002-2003 epidemic. Philos. Trans. R. Soc. B-Biol. Sci..

[6] Yang X (2020). Clinical course and outcomes of critically ill patients with SARS-CoV-2 pneumonia in Wuhan, China: a single-centered, retrospective, observational study. Lancet Respiratory Med..

[7] Fadini GP, Morieri ML, Longato E, Avogaro A (2020). Prevalence and impact of diabetes among people infected with SARS-CoV-2. J. Endocrinol. Investig..

[8] Myers LC, Parodi SM, Escobar GJ, Liu VX (2020). Characteristics of hospitalized adults with COVID-19 in an integrated health care system in California. Jama-J. Am. Med. Assoc..

[9] Barron E (2020). Associations of type 1 and type 2 diabetes with COVID-19-related mortality in England: a whole-population study. Lancet Diabetes Endocrinol..

[10] Krausgruber T (2020). Structural cells are key regulators of organ-specific immune responses. Nature.

[11] Kennerdell JR, Carthew RW (1998). Use of dsRNA-mediated genetic interference to demonstrate that frizzled and frizzled 2 act in the wingless pathway. Cell.

[12] Fire A (1998). Potent and specific genetic interference by double-stranded RNA in *Caenorhabditis elegans*. Nature.

[13] Bartel DP (2009). MicroRNAs: target recognition and regulatory functions. Cell.

[14] Lecellier CH (2005). A cellular MicroRNA mediates antiviral defense in human cells. Science.

[15] Otsuka M (2007). Hypersusceptibility to vesicular stomatitis virus infection in Dicer1-deficient mice is due to impaired miR24 and miR93 expression. Immunity.

[16] Liu X (2019). Initial function of microRNAs as a defence mechanism against invading organisms. ExRNA.

[17] Chen X (2008). Characterization of microRNAs in serum: a novel class of biomarkers for diagnosis of cancer and other diseases. Cell Res..

[18] Zhang Y (2010). Secreted monocytic miR-150 enhances targeted endothelial cell migration. Mol. Cell..

[19] Zhang J (2015). Exosome and exosomal microRNA: trafficking, sorting, and function. Genomics Proteom. Bioinforma..

[20] Valadi H (2007). Exosome-mediated transfer of mRNAs and microRNAs is a novel mechanism of genetic exchange between cells. Nat. Cell Biol..

[21] Yin Y (2014). Tumor-secreted miR-214 induces regulatory T cells: a major link between immune evasion and tumor growth. Cell Res..

[22] Colberg SR (2016). Physical activity/exercise and diabetes: a position statement of the American Diabetes Association. Diabetes Care..

[23] Lin X (2015). Effects of exercise training on cardiorespiratory fitness and biomarkers of cardiometabolic health: a systematic review and meta-analysis of randomized controlled trials. J. Am. Heart Assoc..

[24] Cui SF (2016). Similar responses of circulating microRNAs to acute high-intensity interval exercise and vigorous-intensity continuous exercise. Front. Physiol..

[25] Cui SF (2015). Acute responses of circulating microRNAs to low-volume sprint interval cycling. Front. Physiol..

[26] Yin X (2020). Regulation of circulatory muscle-specific microRNA during 8 km run. Int. J. Sports Med..

[27] Zhou Z (2020). Decreased HD-MIR2911 absorption in human subjects with the SIDT1 polymorphism fails to inhibit SARS-CoV-2 replication. Cell Discov..

[28] Zhou L-K (2020). Absorbed plant MIR2911 in honeysuckle decoction inhibits SARS-CoV-2 replication and accelerates the negative conversion of infected patients. Cell Discov..

[29] Kalarikkal SP, Sundaram GM (2021). Edible plant-derived exosomal microRNAs: exploiting a cross-kingdom regulatory mechanism for targeting SARS-CoV-2. Toxicol. Appl. Pharmacol..

[30] Schultz IC, Bertoni APS, Wink MR (2021). Mesenchymal stem cell-derived extracellular vesicles carrying miRNA as a potential multi target therapy to COVID-19: an in silico analysis. Stem Cell Rev. Rep..

[31] Baar K (2014). Using molecular biology to maximize concurrent training. Sports Med..

[32] Doma K, Deakin GB, Bentley DJ (2017). Implications of impaired endurance performance following single bouts of resistance training: an alternate concurrent training perspective. Sports Med..

[33] Wang Y (2019). Injured liver-released miRNA-122 elicits acute pulmonary inflammation via activating alveolar macrophage TLR7 signaling pathway. Proc. Natl Acad. Sci. USA.

[34] Shao H (2018). New technologies for analysis of extracellular vesicles. Chem. Rev..

[35] Witwer KW (2013). Standardization of sample collection, isolation and analysis methods in extracellular vesicle research. J. Extracell. Vesicles.

[36] Liu Y (2017). miR-19a promotes colorectal cancer proliferation and migration by targeting TIA1. Molecular Cancer..

[37] Wang M (2020). Remdesivir and chloroquine effectively inhibit the recently emerged novel coronavirus (2019-nCoV) in vitro. Cell Res..

