# Supplementary material for: Decreased inhibition of exosomal miRNAs on SARS-CoV-2 replication underlies poor outcomes in elderly people and diabetic patients
Source: Signal Transduct Target Ther. 2021 Aug 11;6:300. doi: 10.1038/s41392-021-00716-y (PMC8355568; doi:10.1038/s41392-021-00716-y)
Supplement: Supplementary file 1 — Supplementary Materials [file 41392_2021_716_MOESM1_ESM.docx]

Supplementary Materials for

Decreased inhibition of exosomal miRNAs on SARS-CoV-2 replication underlies poor outcomes in elderly people and diabetic patients

Yanbo Wang^1,†^, Xiaoju Zhu^1†^, Xia-Ming Jiang^2†^, Jingwei Guo^1†^, Zheng Fu^1†^, Zhen Zhou^1,†^, Ping Yang^3^, Hongyuan Guo^1^, Xu Guo^1^, Gaoli Liang^1^, Ping Zeng^4^, Gengfu Xiao^2^, Jizheng Ma^5^, Xin Yin^1^, Lei-Ke Zhang^2,^*, Chao Yan^1,^*, Chen-Yu Zhang^1,^*

Correspondence to: C.-Y.Z. (Email: cyzhang@nju.edu.cn; Tel: 86-25-89680245; Fax: 02583596234), C.Y. (yanchao@nju.edu.cn) or L.-K.Z. (zhangleike@wh.iov.cn).

**This PDF file includes:**

Tables S1 to S3

Captions for Table S4

**Other Supplementary Materials for this manuscript include the following:**

Table S4

|  | Cohort 1 | | | Cohort 2 | | | Cohort 3 | |
| --- | --- | --- | --- | --- | --- | --- | --- | --- |
| Group name | Young | Old | Healthy | Young | Old | Healthy | Control | Exercise |
| Number (n) | 15 | 15 | 15 | 20 | 20 | 20 | 18 | 18 |
| Age (years) | 20.5±0.5 | 76.5±10.9 | 48.0±6.5 | 20.3±1.2 | 72.5±9.6 | 49.6±9.2 | 20.3±1.9 | 20.6±1.9 |
| Gender (Men) | 7  (46.7%) | 8  (53.3%) | 10  (66.7%) | 8 (40%) | 9  (45%) | 10 (50.0%) | 18 (100%) | 18 (100%) |
| Diabetes | 0 | 0 | 0 | 0 | 0 | 0 | 0 | 0 |
| Hypertension | 0 | 2 (13.3%) | 0 | 0 | 3 (15.0%) | 0 | 0 | 0 |
| Cardiovascular disease | 0 | 4 (26.7) | 0 | 0 | 2 (10.0%) | 0 | 0 | 0 |
| 8-week training programme | N/A | N/A | N/A | N/A | N/A | N/A | No | Yes |

**Table S1.** T**he general characteristics of all the participants.**

**Table S2. Clinical** **characteristics of diabetic patients.**

|  | **Cohort 1** | **Cohort 2** |
| --- | --- | --- |
| Number (n) | **15** | **20** |
| Age (years) | 51.0±19.8 | 54.2±20.1 |
| Gender (Men) | 8 (53.3%) | 10 (50.0%) |
| BMI (kg/m2) | 28.5±4.2 | 29.4±3.7 |
| Diabetes duration (years) | 4.2±3.0 | 3.9±2.8 |
| HbA1c (%) | 9.2±3.3 | 9.5±3.0 |
| GLU(mmol/L) | 10.0±3.6 | 8.7±2.4 |
| OGTT 2h (mmol/L) | 14.4±5.5 | 14.6±3.5 |
| Ischemic heart disease | 0 | 1 (5%) |
| TC | 4.7±1.4 | 5.4±3.0 |
| TG | 1.8±0.8 | 3.5±8.5 |

**Table S3. Summary of the training programs**

|  | **Monday** | **Tuesday** | **Wednesday** | **Thursday** | **Friday** | **Saturday** | **Sunday** |
| --- | --- | --- | --- | --- | --- | --- | --- |
| **A.m.** | Cardiorespiratory exercise training | Muscular strength or endurance | Cardiorespiratory exercise training | Muscular strength or endurance | Cardiorespiratory exercise training | Muscular strength or endurance | Rest |
| **P.m.** | Anaerobic exercises | Plyometric | Anaerobic exercises | Plyometric exercises | Anaerobic exercises | Plyometric exercises | Rest |

Table S4. (Separate file) Bioinformatics prediction of the interaction between differentially expressed miRNA in the young group and SARS-CoV-2 genome.
